# Effect of yoga on cancer-related fatigue in patients with breast cancer: A systematic review and meta-analysis

**DOI:** 10.1097/MD.0000000000036468

**Published:** 2024-01-05

**Authors:** Lingyu Hou, Jianhua Wang, Meina Mao, Zerui Zhang, Dandan Liu, Shun Gao, Kaixue Liang, Linlin Lu

**Affiliations:** a Peking University Shenzhen Hospital, Shenzhen, People’s Republic of China; b Medical School, Weifang University of Science and Technology, Shandong, Weifang, People’s Republic of China.

**Keywords:** breast cancer, cancer-related fatigue, meta-analysis, yoga

## Abstract

**Background:**

Breast cancer is a common malignant tumor in women and most patients with breast cancer experience fatigue. Numerous studies have investigated the relationship between yoga and cancer-related fatigue (CRF) in patients with breast cancer. However, these studies drew their conclusions from small sample sizes and lacked sufficient evidence to demonstrate that yoga can effectively alleviate CRF. Therefore, this meta-analysis aims to systematically examine the effects of yoga on cancer fatigue in patients with breast cancer and establish a scientific basis for enhancing their quality of life.

**Objective:**

To assess the effect of yoga on CRF in patients with breast cancer.

**Methods:**

Computer searches were conducted on PubMed, Embase, Web of Science, CKNI, and Wanfang databases to retrieve articles related to yoga and CRF in patients with breast cancer from the hospital establishment date to July 2023. The literature was independently screened, and the information was extracted by the researchers. A meta-analysis was conducted using Review Manager Software (version 5.3).

**Results:**

The findings from the meta-analysis of 18 studies indicate that yoga can effectively enhance CFR (standardized mean difference (SMD) = −0.51, 95% confidence interval [CI] = −0.92 to −0.10), improve sleep quality (MD = −3.86, 95%CI = −4.03 to −3.70) in patients with breast cancer, alleviate anxiety and depression (SMD = −0.93, 95%CI = −1.68, −0.18, SMD = −1.23, 95%CI = −2.02 to −0.44), and enhance quality of life (MD = −11.20, 95%CI = −14.16 to −8.24).

**Conclusion:**

Our study offers evidence for the subsequent reduction of CFR in patients with breast cancer. Yoga can alleviate fatigue, improve sleep quality and negative emotions, and improve the quality of life of patients with breast cancer.

## 1. Introduction

Breast cancer is the most common cancer among women worldwide, accounting for 25.1% of all cancers.^[1]^ In 2012, an estimated 1.67 million new cases of breast cancer and 520,000 breast cancer-related deaths were reported worldwide. It is anticipated that by 2021, cancer incidence will increase to 85 cases per 100,000 women.^[2]^ Delayed or untreatable treatment for patients with cancer increases their health burden.^[3]^ Nevertheless, in recent years, the emergence of neoadjuvant therapy has increased treatment probability and improved the survival rate of patients. However, survivors also face a series of physical and mental issues, including premature menopause, body image disorder, fatigue, and depression.^[4–6]^ Cancer-related fatigue (CRF) is a common symptom in patients with breast cancer.^[7]^ Related to cancer or cancer treatment, CRF is a painful and persistent subjective state characterized by subjective feelings of physical, emotional, or cognitive fatigue or exhaustion inconsistent with recent activity levels. It negatively affects and disrupts daily activities of the patients.^[8]^ CRF is more severe and long-lasting than typical fatigue and cannot be alleviated by rest or sleep. Consequently, recent research has been focused on improving CRF in patients with breast cancer. However, there is a lack of standard protocols for treating fatigue.^[9]^ During COVID-19, advancements in tablets, online courses, artificial intelligence, and social media have transformed teaching and learning methods. For patients with breast cancer, using the Internet for exercise can protect their health and enable them to learn at their convenience and pace.^[10]^

Yoga employs a series of specific body postures to attain health and relaxation, which not only alleviates psychological stress and prevents and treats mental illness but also improves body form and function and effectively prevents and treats chronic diseases, including cardiovascular and cerebrovascular diseases.^[11]^ Yoga exercise is simple and has been extensively performed in both online and offline settings. In recent years, the effectiveness of yoga on relieving fatigue in patients with breast cancer has attracted significant interest. Several studies investigating the effects of yoga on CRF in patients with breast cancer have reported both positive and negative findings.^[12]^ Dong only investigated the impact of yoga on CRF in patients with breast cancer, leaving the impact on sleep quality unclear.^[12]^ This study comprehensively gathered the literature from randomized controlled studies involving yoga intervention in patients with breast cancer. The impact of these interventions was verified through meta-analysis, providing authoritative, reliable, and comprehensive evidence to support clinical nursing practices for patients with breast cancer and establish an evidence-based foundation for medical care decision-making.

## 2. Methods

### 2.1. Literature search

This meta-analysis followed the guidelines outlined in the Preferred Reporting Program for Systematic Review and Meta-Analyses. Multiple databases, including PubMed, Web of Science, Cochrane Library, CNKI, Wan Fang Data, and VIP, were thoroughly searched by 2 independent researchers. The search period spanned from the establishment of the library until July 2023. The search is restricted to articles published in Chinese or English. The following keywords, including “Breast Neoplasm,” “Neoplasm, Breast,” “Breast Tumors,” “Breast Tumor,” “Tumor, Breast,” “Tumors, Breast,” “Neoplasms, Breast,” “Breast Cancer,” “Cancer, Breast,” “Mammary Cancer,” “Cancer, Mammary,” “Cancers, Mammary,” “Mammary Cancers,” “Malignant Neoplasm of Breast,” “Breast Malignant Neoplasm,” “Breast Malignant Neoplasms,” “Malignant Tumor of Breast,” “Breast Malignant Tumor,” “Breast Malignant Tumors,” “Cancer of Breast,” “Cancer of the Breast,” “Fatigue,” “Lassitude,” “Yoga,” and “Yogic,” were employed for the searches.

### 2.2. Inclusion and exclusion criteria

Research type: Randomized controlled trial.

Participants: The subjects were individuals with a definite pathological or cytological diagnosis of breast cancer.

Intervention measure: Patients performed different types of yoga.

#### 2.2.1. Outcome.

CRF score: measures sleep quality, anxiety, depression, and quality of life.

#### 2.2.2. Exclusion criteria.

Non-Chinese and English literature, duplicate publications, inability to obtain the required data, and index results meeting the literature requirements.

#### 2.2.3. Data extraction.

Data from all eligible studies were independently extracted by 2 authors (LYH and JHW). The following variables were removed from each study: first author name, publication year, country, study design, sample size, age, sex ratio, and study quality score. Any disagreements were resolved through discussion or by consulting a senior reviewer.

#### 2.2.4. Quality assessment.

The risk of bias in the included randomized controlled trials was independently evaluated by 2 reviewers using the Cochrane assessment tool, which comprises 7 domains: adequate sequence generation, allocation concealment, blinding of participants and personnel, blinding of outcome assessment, incomplete outcome data, selective reporting, and other bias.

#### 2.2.5. Data synthesis and analysis.

Review Manager Software (version 5.3) was used to synthesize the data from the included studies. If *P* > 0.1 and *I*^2^ < 50%, a fixed-effects model was chosen for the analysis because of the homogeneity of the studies. Conversely, if *P* < 0.1 and *I*^2^ ≥ 50%, a random-effects model was used. A descriptive analysis was conducted without a meta-analysis if *P* < 0.1 and the sources of heterogeneity were unknown. The weighted mean difference and 95% confidence interval (CI) were calculated for individual trials when dealing with continuous data. When the outcome assessment tools were different, the standardized mean difference (SMD) was used. Sensitivity analysis approaches, such as individual exclusion and observation, were used to examine the sources of clinical heterogeneity. A random-effects model was used if there was no clinical heterogeneity. If significant clinical heterogeneity was observed, subgroup analysis was used. Publication bias was determined using funnel plot.

## 3. Results

### 3.1. Study selection and baseline characteristics

A total of 1752 results were generated from the literature search, with most being excluded either as duplicates or due to their lack of relevance to our meta-analysis. Subsequently, the full text of 58 articles was reviewed. Finally, this study included 18 studies.^[13–30]^ See the ***Flowchart***. Table 1 ***shows the characteristics of the study and the participants.***

**Table 1:** The characteristics of the study and participants

Author	Year	Country	Sample	Age	BMI	Weight	Tumor staging	Intervention time	Frequency	Outcome	Quality evaluation
Liu^[13]^	2022	China	Yoga:61 CG:62	NA	24.71 ± 3.69/23.69 ± 3.16	NA	I–II	8 weeks	90 min/week	PFS-R/HADS-D/FACT-B	A
Moadel^[14]^	2007	USA	Yoga:108 CG:65	55.11 ± 10.07/54.23 ± 9.81	NA	NA	I–IV	12 weeks	12 sessions/week	FACIT-Fatigue	B
Bower^[15]^	2012	USA	Yoga:16 CG:15	54.4 ± 5.7/53.3 ± 4.9	NA	NA	0–II	12 weeks	2 weeks/time, 90 min/time	FSI/PSQI/ BDI-II	A
Vadiraja^[16]^	2017	India	Yoga:33 CG:31	50.54 ± 8.53	NA	NA	NA	3 months	NA	FSI	B
Cramer^[17]^	2015	Germany	Yoga:19 CG:21	48.3 ± 4.8/50.0 ± 6.7	NA	69.8 ± 11.9/74.3 ± 17.0	I–III	12 weeks	90 min/week	FACIT-F/HADS-A/HADS-D/FACT-B	A
Vadiraja^[18]^	2009	India	Yoga:44 CG:44	NA	NA	NA	II–III	6 weeks	3 times/week	EORTCQLQ-C30-Fatigue	B
Chaoul^[19]^	2018	USA	Yoga:74 CG:85	49.5 ± 9.8/50.4 ± 10.3/49 ± 10.1	NA	NA	I–III	12 weeks	4 sessions/week, 75–90 min/time	BFI/PSQI	B
Lötzke^[20]^	2016	Germany	Yoga:45 CG:47	51.0 ± 11.0/51.4 ± 11.1	NA	NA	NA	12 weeks	60 min/week	EORTCQLQ-C30-Fatigue	B
Rahmani^[21]^	2015	Iran	Yoga:12 CG:12	43.25 ± 3.07/44.8 ± 3.28	NA	NA	l–III	8 weeks	Once/week, 2 h/time	QLQ-C30-Fatigue	B
Jong^[22]^	2018	Netherlands	Yoga:47 CG:36	51 ± 8/51 ± 7.3	NA	NA	I–III	12 weeks	Once/week	EORTCQLQ-C30F/HADS-D	A
Wang^[23]^	2014	China	Yoga:40 CG:42	18~60	NA	NA	NA	4 months	4 times/week, 1 time/day, 50 min/time	CFS	B
Zeng^[24]^	2017	China	Yoga:24 CG:23	NA	NA	NA	NA	4 months	2 days/once, once/30 min	CFS	B
Yu^[25]^	2021	China	Yoga:59 CG:59	38.8 ± 10.9/39.5 ± 10.3	NA	NA	II–III	8 weeks	Twice/day, 3–5 times/week	CFS	B
Cai^[26]^	2022	China	Yoga:36 CG:37	51.17 ± 8.79/	24–27.9	NA	I–III	21 days	NA	PFS-R/SDS/SAS	B
Danhauer^[27]^	2009	USA	Yoga:13 CG:14	54.3 ± 9.6/57.2 ± 10.2	NA	NA	I–IV	10 weeks	75min	FACT-f/FACT-B	
Li^[28]^	2019	China	Yoga:45 CG:45	47.5 ± 8.2/46.7 ± 9.5	NA	NA	NA	2 months	3 times/week, 60 min/time	PSQI	B
Banerjee^[29]^	2007	Singapore	Yoga:35 CG:23	NA	NA	NA	II–III	6 weeks	NA	HADS-A/HADS-D	B
Jin^[30]^	CA	China	Yoga:50 CG:50	55–73	NA	NA	NA	16 weeks	1 h/time, 3 times/week	SDS/SAS/FACT-B	B

BDI = beck depression inventory, BFI = brief fatigue inventory, CFS = cancer fatigue scale, FSI = fatigue symptom inventory, HADS = Hospital Anxiety and Depression Scale, PSQI = Pittsburgh sleep quality index, SAS = self-rating anxiety scale, SDS = self-rating depression scale, FACT-B = The Functional Assessment of Cancer Therapy—Breast, FACIT-F = functional assessment of chronic illness therapy-fatigue, PFS-R = The Revised Piper Fatigue Scale.

### 3.2. Results of the meta-analysis

#### 3.2.1. Cancer-related fatigue.

**Figure 1.**
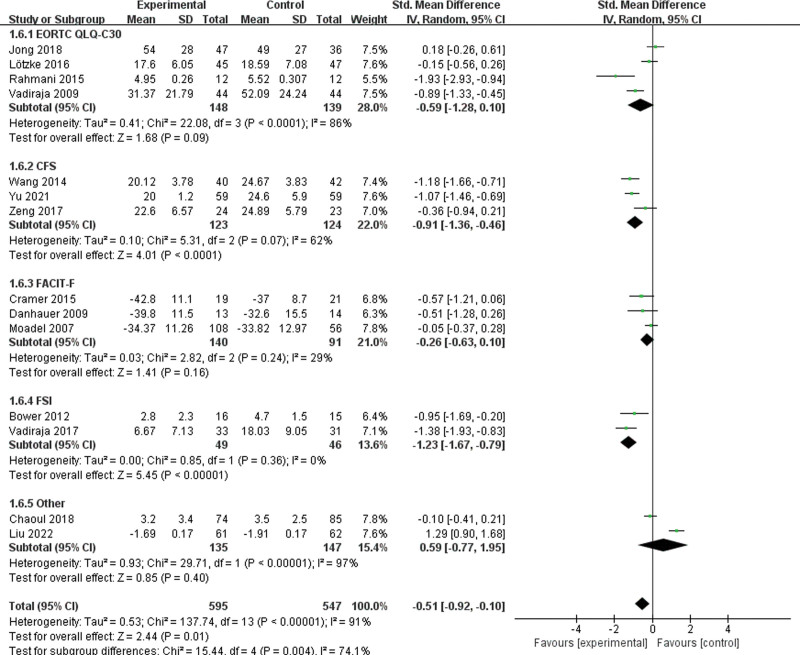
The effect of yoga on CRF. CRF = cancer-related fatigue.

CRF was described in 14 studies. A random-effects model was used for data synthesis because of significant heterogeneity in the findings (*I*^2^ = 91%, *P* < 0.001). The findings indicated that the yoga group was beneficial for relieving CRF (SMD = −0.51, 95%CI = −0.92 to −0.10, Z = 2.44, *P* = 0.01). Sensitivity analysis was conducted to identify the potential sources of heterogeneity. No changes in the findings were observed even after excluding all studies, indicating the stability of the findings. Subgroup analysis was then performed based on the application of different scales, and the following groups were identified: the EORTC QoL C30 group (SMD = −0.59, 95%CI = −1.28 to 0.10), the cancer fatigue scale group (SMD = −0.91, 95%CI = −1.36 to −0.46), the functional assessment of chronic illness therapy-fatigue group (SMD = −0.26, 95%CI = −0.63 to 0.10), the fatigue symptom inventory group (SMD = −1.23, 95%CI = −1.67 to −0.79), and other study type groups (SMD = 0.59, 95%CI = −0.77,1.95) (Fig. 1).

#### 3.2.2. Pittsburgh sleep quality index.

**Figure 2.**
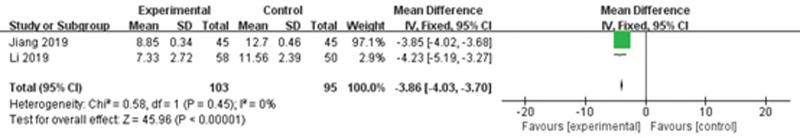
The effect of yoga on PSQI. PSQI = Pittsburgh sleep quality index.

Four studies reported findings on sleep quality. Significant heterogeneity was observed among the studies (*I*^2^ = 89%, *P* < 0.0001), and sensitivity analysis was performed. Two articles were excluded, and the data were synthesized using a fixed-effect model (*I*^2^ = 0, *P* = 0.45). The meta-analysis results indicated that yoga was more effective in enhancing sleep quality (MD = −3.86, 95%CI = −4.03 to −3.70, Z = 45.96, *P* < 0.0001) (Fig. 2).

#### 3.2.3. Anxiety.

**Figure 3.**
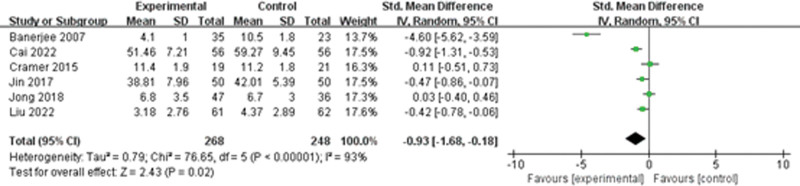
The effect of yoga on anxiety.

Six studies discussed anxiety, and a random-effects model was used to synthesize the data due to significant heterogeneity in the findings (*I*^2^ = 93%, *P* < 0.001). The findings indicated that yoga was beneficial for relieving anxiety (SMD = −0.93, 95%CI = −1.68 to −0.18, Z = 2.43, *P* = 0.02). Sensitivity analysis was conducted to identify the potential sources of heterogeneity. Even after excluding all studies, the findings remained unchanged, demonstrating that the findings were stable. Subsequently, subgroup analysis was conducted based on the application of different scales, sources of reference, and year of publication, but significant heterogeneity persisted (Fig. 3).

#### 3.2.4. Depression.

**Figure 4.**
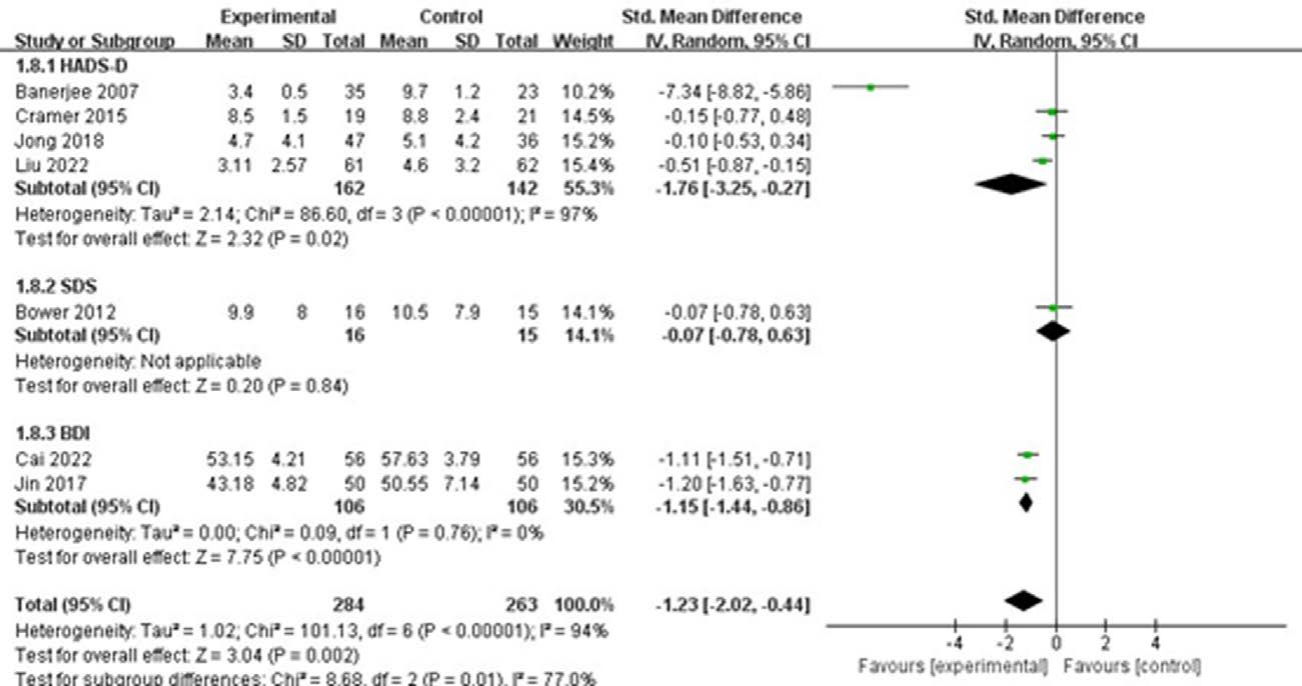
The effect of yoga on depression.

Depression was addressed in 7 studies. A random-effects model was used for data synthesis due to significant heterogeneity in the results (*I*^2^ = 94%, *P* < 0.001). The findings indicated that yoga had a positive impact on reducing CRF (SMD = −1.23, 95%CI = −2.02 to −0.44, Z = 3.04, *P* = 0.002). A sensitivity analysis was conducted to identify potential sources of heterogeneity. Even after excluding all studies, the findings remained unchanged, indicating that the results were stable. Subsequently, subgroup analysis was conducted based on the application of different scales, revealing significant effects in the Hospital Anxiety and Depression Scale-D group (SMD = −1.76, 95%CI = −3.25 to −0.27) and beck depression inventory (BDI) group (SMD = −1.15, 95%CI = −1.44 to −0.86) (Fig. 4).

#### 3.2.5. Quality of life.

**Figure 5.**
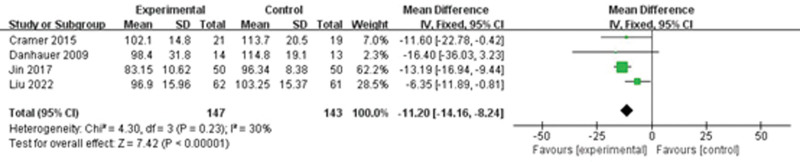
The effect of yoga on quality of life.

Four studies described the quality of life. A fixed-effect model was used for data synthesis since the results showed no significant heterogeneity (*I*^2^ = 30%, *P* = 0.23). The findings indicated that the yoga group improved the quality of life (MD = −11.20, 95%CI = −14.16 to −8.24, Z = 7.42, *P* < 0.00001) (Fig. 5).

#### 3.2.6. Publication bias.

**Figure 6.**
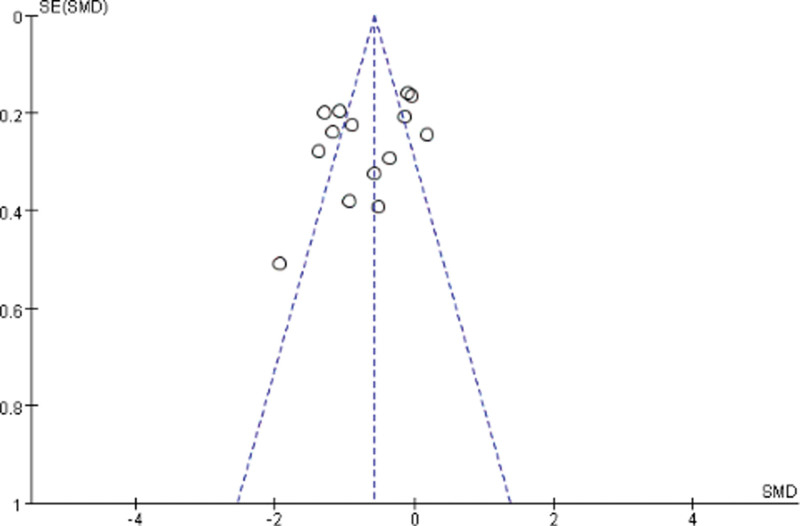
Funnel plot.

Publication bias was evaluated using funnel plots. In this study, only CRF was evaluated for publication bias. Generally, the roughly symmetrical graph indicated no publication bias (Fig. 6).

## 4. Discussions

CRF is one of the most common symptoms in patients with breast cancer, with more than half experiencing it during or after treatment. Fatigue can result in decreased quality of life, treatment compliance, and even worse survival outcomes than patients unaffected by fatigue. The widely accepted exercise regimen for patients with breast cancer is approximately 150 minutes of moderate-to-vigorous aerobic exercise per week, but only 30% to 47% of patients with breast cancer exercise adhere to this guideline.^[12]^ Yoga is a program of physical and mental exercises that includes physical postures, controlled breathing, and meditation. The primary objective of yoga classes is the prevention of fatigue, and similar to aerobic exercise training, they can also improve the physical function of patients. Currently, many major cancer treatment centers worldwide incorporate yoga as an adjunct therapy.^[12]^ A previous meta-analysis also revealed the positive impact of yoga on fatigue in patients with breast cancer.^[31]^ This analysis neither conducted a subgroup analysis based on a measurement tool for fatigue nor investigated the effect on sleep quality in patients with breast cancer. Seventeen articles were examined, and high heterogeneity was observed in the case of CFR. To further investigate whether yoga can improve CFR, sensitivity and subgroup analyses were conducted. Furthermore, our study investigated the effects of yoga on sleep quality, anxiety, and depression in patients with breast cancer.

This study established that yoga can effectively improve CRF in patients with breast cancer. These findings are consistent with those reported by Dong et al^[12]^ Conversely, these findings are inconsistent with the research results of Liu,^[13]^ primarily due to the longer duration of yoga exercise in the literature included in this study, resulting in a more significant effect. CRF, a common symptom in patients with cancer, can result in reduced quality of life, reduced treatment compliance, and even worse survival than in patients unaffected by fatigue.^[32]^ Numerous leading cancer centers around the world incorporate yoga as an adjunct therapy. Yoga is a gentle, safe aerobic exercise that includes body postures, controlled breathing, and meditation that can enhance a patient physical function. Yoga exercise increases the output of the human heart, thereby boosting the oxygen content in the blood and improving the physical and emotional well-being of patients with cancer, ultimately relieving physical fatigue.

Patients with breast cancer experience concerns related to the disease, physiological changes resulting from treatment, chemotherapy-induced side effects, and psychological stress. Therefore, they have various sleep problems, including challenges in falling asleep, low sleep efficiency, and daytime fatigue. Sleep disorders are prevalent in 51% to 90% of cancer survivors.^[12]^ Currently, there are only few meta-analyses addressing the effect of yoga on sleep quality. The summary of this study indicates that yoga can effectively improve the sleep quality of patients with breast cancer. Yoga practice involves rhythmic whole-body muscle relaxation exercises that reduce skeletal muscle tension, decrease arousal levels in the cerebral cortex, reduce oxygen consumption, and improve sleep.^[33]^ In addition, abdominal deep breathing techniques are used to increase the oxygen content of the chest, activate the parasympathetic nerve, and maintain a state of calmness, thereby reducing psychological pressure and promoting sleep.^[34]^

Yoga can effectively alleviate anxiety, tension, and other negative emotions experienced by patients with breast cancer by freeing their bodies and minds. Yoga involves gentle aerobic exercise in which the nervous system generates micro-electrical stimulation to relax the cerebral cortex and gradually relieve muscle tension, anxiety, depression, and other negative emotions.^[35]^ In addition, yoga training is conducted collectively, enabling patients to engage in communication, improve their confidence in overcoming the disease, and alleviate anxiety, depression, and other negative emotions.^[36]^ This study further demonstrates the effectiveness of yoga in reducing anxiety and depression in patients with breast cancer. Furthermore, yoga improves the quality of life of patients with breast cancer by reducing fatigue and improving sleep quality.

## 5. Recommendation

Our study demonstrates that yoga offers certain advantages in enhancing CRF and sleep quality in patients with breast cancer. However, in the process of yoga exercise for patients with breast cancer, consultations with a doctor or professional health instructor should be considered based on their condition to ensure that their physical condition is appropriate for yoga exercise. Initiation of gentle yoga exercises and gradual increase in difficulty and challenge are advised. Overexertion or the adoption of unsuitable positions should be avoided. Adequate rest is also essential. Furthermore, yoga place importance on not only physical exercise but also mental balance and inner peace.

## 6. Limitations and future implications

Although a comprehensive review of the literature on breast cancer fatigue was conducted, this study has some limitations. The assessment tools for the outcome indicators in each analysis differ, leading to a certain degree of heterogeneity. Furthermore, each study varies in terms of intervention time, frequency, and specific implementation methods of intervention measures, resulting in some heterogeneity. This study included only the Chinese and English literature, which may have introduced bias to the results. Finally, factors such as sample size, location, research method, and variables or conditions may have some impacts on the findings of this study.

## 7. Conclusion

Our pooled findings demonstrated that the use of yoga improved CFR, sleep quality, anxiety and depression, and quality of life in patients with breast cancer. Yoga can be promoted for patients with breast cancer. However, during yoga exercises, the patients must be within safe limits. To obtain definitive conclusions on the effectiveness of yoga for patients with breast cancer, well-designed studies with a larger sample size and more detailed outcome indicators are needed, considering certain limitations in this meta-analysis.

## Author contributions

**Data curation:** Lingyu Hou, Jianhua Wang, Meina Mao, Shun Gao, Kaixue Liang.

**Investigation:** Lingyu Hou.

**Software:** Lingyu Hou, Jianhua Wang, Meina Mao, Zerui Zhang.

**Writing–original draft:** Lingyu Hou, Meina Mao, Zerui Zhang.

**Writing – review & editing:** Dandan Liu, Linlin Lu.
